# Optimization strategies for metabolic networks

**DOI:** 10.1186/1752-0509-4-113

**Published:** 2010-08-13

**Authors:** Alexandre Domingues, Susana Vinga, João M Lemos

**Affiliations:** 1 INESC-ID - R. Alves Redol 9, 1000-029 Lisboa, Portugal; 2 FCM-UNL - C Mártires Pátria 130, 1169-056 Lisboa, Portugal; 3 IST-UTL - Avenida Rovisco Pais, 1000 Lisboa, Portugal

## Abstract

**Background:**

The increasing availability of models and data for metabolic networks poses new challenges in what concerns optimization for biological systems. Due to the high level of complexity and uncertainty associated to these networks the suggested models often lack detail and liability, required to determine the proper optimization strategies. A possible approach to overcome this limitation is the combination of both kinetic and stoichiometric models. In this paper three control optimization methods, with different levels of complexity and assuming various degrees of process information, are presented and their results compared using a prototype network.

**Results:**

The results obtained show that Bi-Level optimization lead to a good approximation of the optimum attainable with the full information on the original network. Furthermore, using Pontryagin's Maximum Principle it is shown that the optimal control for the network in question, can only assume values on the extremes of the interval of its possible values.

**Conclusions:**

It is shown that, for a class of networks in which the product that favors cell growth competes with the desired product yield, the optimal control that explores this trade-off assumes only extreme values. The proposed Bi-Level optimization led to a good approximation of the original network, allowing to overcome the limitation on the available information, often present in metabolic network models. Although the prototype network considered, it is stressed that the results obtained concern methods, and provide guidelines that are valid in a wider context.

## Background

Current metabolic engineering processes allow to manipulate metabolic networks to improve the desired characteristics of biochemical systems [1]. These manipulations may lead to the maximization of the normal product yield or redirect the production to a flux that was residual or non-significant in the original network. The high level of uncertainty in metabolic network models knowledge makes it extremely difficult to determine what are the required manipulations needed to attain a given objective. Since an heuristic approach to such problems does not allow to explore the maximum potential of metabolic engineering, two approaches are usually considered when modeling metabolic networks. Kinetic models describe the complete dynamics of the network, and have proven useful to implement optimization and control over the network, such as in [2]. The creation of reliable kinetic models involves the estimation of parameters, the complexity of this task increasing with the size of the network considered.

The second approach models the networks on the basis of reaction stoichiometry. Although easier to obtain, these models lack the ability to directly predict the dynamics of the system.

Several techniques have been proposed to optimize and infer network characteristics from these models. In [3] a platform that combines many of these methods is presented. Flux Balance Analysis (FBA) allows the determination of the optimal flux distribution on a network described in terms of the stoichiometry of the reactions and yields reliable results in the study of metabolic systems [4-6]. A review of the method can be seen in [7].

When optimizing a metabolic network for a given objective two distinct problems must be addressed. The first is to find which branch or branches must be manipulated. The second is to determine what type of alterations must be done. Strategies such as OptKnock [8] and the work in [9] address the first problem. In this work a strategy for the second problem is described.

The simulation and engineering of metabolic network models typically involves complex optimization procedures. Geometric Programming (GP), one of the techniques used in this paper, is a powerful mathematical optimization tool that can be applied to problems where the objective and constraint functions have a special form [10]. GP is of particular interest because it can solve large scale problems with extreme efficiency and reliability [11]. Furthermore it has been shown that a problem formulated in S-Systems form can be solved with GP after a minimum adaptation [12].

A common optimization problem is the maximization of the final concentration of a metabolite whose formation competes with the natural objective of the cell (*e.g*. maximization of biomass). In this work, a prototype network with such behavior is taken as example and the corresponding optimization problem is solved with three alternative methods.

It is stressed that the emphasis of this work is on the methods and not the specific network considered. The key point of the paper consists in establishing properties of a number of optimization methods that may serve as guidelines when considering more complex networks.

This will be further explained in the next section.

## Results and Discussion

An overall view of the problem considered and paper contributions is first presented. Details may then be seen in subsequent sections.

The problem to consider consists in finding a control function, defined over a finite interval of operation time, such that the final concentration of a desired product is maximum. This product is yielded by a metabolic network that, depending on the control function, either produces it or a product that favors cell growth. In order to settle ideas, assume that the control variable *u *is such that it is constrained to be in the interval [0, 1], with *u *= 0 corresponding to only production of cell growth product and no production of the desired product, and *u *= 1 corresponds to the inverse situation. Values of *u *in between 0 and 1 correspond to a mixed production in a way that depends on the network dynamics.

Since the optimization is with respect to a time function, this is an in finite dimensional problem. However we prove in this paper, using Pontryagin's Maximum Principle [13], that the optimal control only assumes values of 0 and 1. This is *a priori *assumed by other authors [14] and receives now a solid justification. It is a result valid for similar metabolic network problems that aim at optimizing a final yield (e.g. a concentration at the end of the optimization time interval, such as in [15]) and such that the control enters linearly in the network equations.

The significance of this result consists in the fact that, instead of searching the optimal control among piecewise continuous functions assuming values between 0 and 1, one only has to look functions assuming the extreme values of 0 and 1.

Furthermore, in the case study considered, it is shown that the optimum has only one switch between 0 and 1. Therefore the search for the optimum is reduced to find the switching instant, *t*_*reg*_, that leads to the maximum final yield. Considering the structure of the metabolic network, this is intuitive: the optimum is achieved by first applying all cell resources to population growth and, after *t*_*reg*_, to redirect them to desired production. If *t*_*reg *_is too small, the desired production rate is higher during more time, but the cell population to which it applies is small. If *t*_*reg *_is too big, there are many cells to produce, but they only act during a small time interval. Hence, there is an optimum value for *t*_*reg*_.

As mentioned in the Background section, a major problem is the high level of uncertainty in the knowledge about metabolic network dynamics. In this respect we consider different optimization algorithms that assume various degrees of information about the system to be optimized.

The first is direct optimization. This assumes complete knowledge about the system and is included to establish a benchmark with which other methods may be compared.

The other two methods are variants of a bi-level algorithm designed in order to accommodate missing information on the network kinetics. Both cases differ from the type of inner-optimization: Geometric Programming in one case and Linear Programming in the other. Both methods lead to good approximations of the optimal control, with a slight advantage of the one relying on Geometric Programming.

### Prototype network model

**Figure 1:**
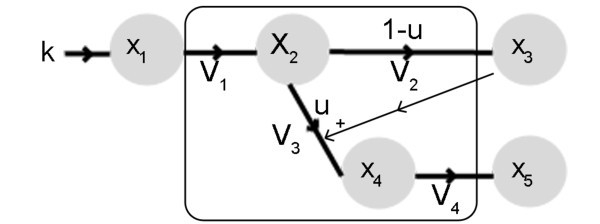
**Prototype network**. The circles correspond to metabolites and the arrows to fluxes with the reaction rates indicated.

The optimization strategies were tested on a prototype network that is a modified version of a previously one suggested in [16]. The choice of this network was due to its widespread use as a test benchmark for several optimization algorithms. A graphical representation of the network is shown in Figure 1 associated with the following set of ordinary differential equations:

(1) $$\begin{array}{l} \frac{dx_{1}}{dt}\;=\;k-v_{1}\\ \frac{dx_{2}}{dt}\;=\;v_{1}-v_{2}(1-u)-v_{3}u\\ \frac{dx_{3}}{dt}=v_{2}(1-u)\\ \frac{dx_{4}}{dt}\;=\;v_{3}u-v_{4}\\ \frac{dx_{5}}{dt}\;=\;v_{4} \end{array}$$

Here the states *x*_*i *_, *i *= 1,..., 5 are metabolite concentrations at the network nodes, *v*_*i*_, *i *= 1,..., 4 are fluxes associated to the metabolic network branches and *k *is a constant parameter that represents the uptake of *x*_1_. In the equations, *u *represents a control function that allows to redirect the flux between the branches *x*_2 _→ *x*_3 _and *x*_2 _→ *x*_4_. Assuming that *x*_3 _represents a precursor of the cellular objective (such as growth) and *x*_5 _the desired product, if *u*(*t*) is biased towards the branch of *v*_2 _this yields the formation of *x*_3 _but little or no production of *x*_5_. If *u*(*t*) is biased towards the branch of *v*_3 _the production of *x*_5 _will be affected by the low concentration of *x*_3 _(since there is a forward feedback). Thus, there is an optimal profile for *u*(*t*) to maximize the concentration of *x*_5 _at the final time *t*_*final*_.

In the framework of S-systems [16] the prototype network is described by:

(2) $$\begin{array}{l} \frac{dx_{1}}{dt}\;=\;k-\beta_{1}x_{1}^{h_{11}}\\ \frac{dx_{2}}{dt}\;=\;\alpha_{2}x_{1}^{g_{21}}-\beta_{2}x_{3}^{h_{23}}x_{2}^{h_{22}}\\ \frac{dx_{3}}{dt}=\alpha_{3}x_{2}^{g_{32}}\left(1-u\right)\\ \frac{dx_{4}}{dt}\;=\;\alpha_{4}x_{3}^{g_{43}}x_{2}^{g_{42}}u-\beta_{4}x_{4}^{h_{44}}\\ \frac{dx_{5}}{dt}\;=\;\alpha_{5}x_{4}^{g_{54}} \end{array}$$

where *β*_*i *_are the rate constants, *g*_*ij *_and *h*_*ij *_are the kinetic orders. Table 1 shows the list of parameters. All the simulations using the prototype network assume *x*(0) = [0.8 0 1 0 0].

**Table 1:** Parameters used in the prototype network.

**Param**.	Value	**Param**.	Value
*α*_2_	8	*h*_11_	0.5

*α*_3_	4.0556	*h*_22_	1.4224

*α*_4_	1.8397	*h*_23_	0.6109

*α*_5_	4.0556	*h*_44_	0.5829

*β*_1_	1	*g*_21_	0.5

*β*_2_	5.1179	*g*_32_	0.4171

*β*_4_	4.0556	*g*_42_	2.8274

k	0.8	*g*_43_	1.4646

		*g*_54_	0.5

### Direct optimization

Direct optimization uses model (2) with the set of parameters from Table 1.

**Figure 2:**
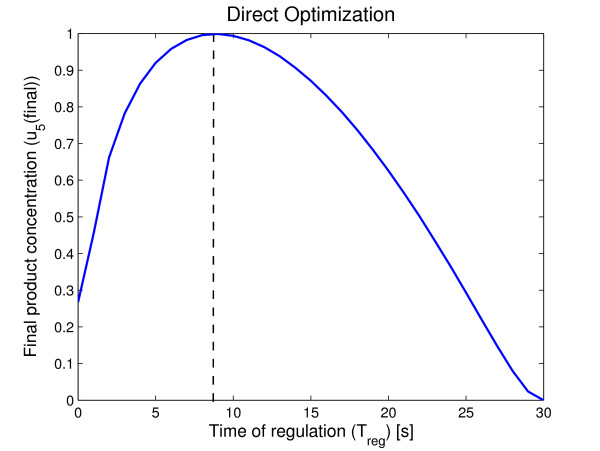
**Results of the simulation using Direct optimization**. The final product concentration is shown as a function of *T*_*reg*_. For the value of *T*_*reg *_corresponding to the dotted line there is a maximum yield.

On a first approach, all possible integer values of *t*_*reg *_in the interval *t*_*reg *_= [1, 30] were used to compute the final product concentration *x*_5_(*t*_*final*_) where *t*_*final *_= 30s. Figure 2 plots the resulting function *J*(*t*_*reg*_) = *x*_5_(*t*_*final*_).

It is clear from Figure 2 that there is an optimal value for the time of regulation that maximizes the yield of *x*_5_. For the network considered, the optimal time of regulation is *t*_*reg *_= 9*s*. If *u*(*t*) switches from 0 to 1 before *t*_*reg *_the formed biomass will not be enough to maximize *x*_5_(*t*_*final*_). On the other hand, if *u*(*t*) switches from 0 to 1 after *t*_*reg*_, there will be more biomass but there time will not be enough time to produce the maximum possible amount of *x*_5_.

**Figure 3:**
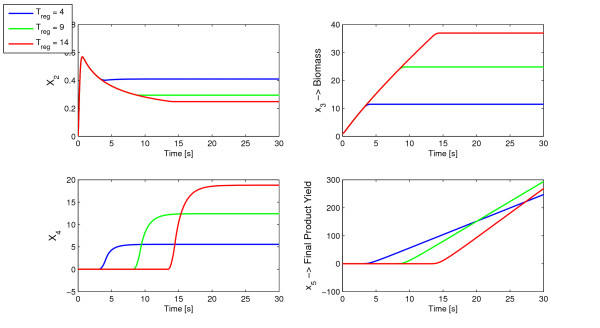
**Comparison of three *u*(*t*) profiles**. Three time profiles for the control function *u*(*t*) (above) and the corresponding product yield (below). The solid line is the optimal *T*_*reg *_obtained by Direct Optimization.

To illustrate better the behavior of the prototype network, simulations were made for *t*_*reg *_= 4, *t*_*reg *_= 9 and *t*_*reg *_= 14. The obtained optimal *t*_*reg *_= 9 is compared in Figure 3 with lower and upper values in order to show the different time evolution of the metabolites.

### Bi-Level Optimization

**Figure 4:**
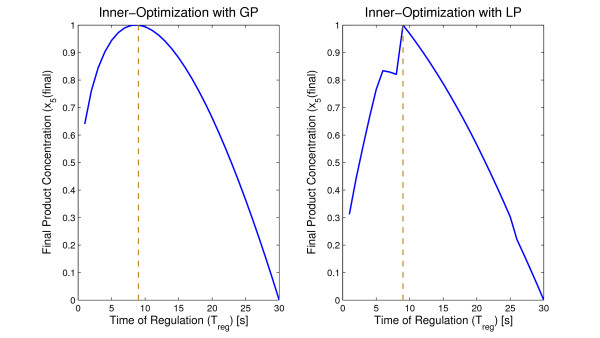
**Result of the optimization using the Inner Optimization with Geometric Programming (left) and Linear Programming (right)**. The profiles of the production of *x*_5 _remain similar to the simulation using Direct optimization.

The Bi-Level optimization was used to test all the possible values of *t*_*reg*_. Figure 4 plots the normalized curves for *J*(*t*_*reg*_) = *x*_5_(*t*_*final*_) for the two optimizations, inner-optimization using Geometric Programming (GP) and inner-optimization using Linear Programming (LP). By comparing Figure 4 with Figure 2 it can be seen that the profiles remain similar. The final product yield, *x*_5_(*t*_*final*_), increases with *t*_*reg *_until the optimal value is reached, then it starts decreasing.

The optimal time of regulation obtained with both GP and LP on the inner optimization was *t*_*reg *_= 9. As shown mathematically in the methods section, the optimal control function is either 0 or 1, provided that the dynamics depends linearly on the control and the cost to optimize has only a final term.

**Figure 5:**
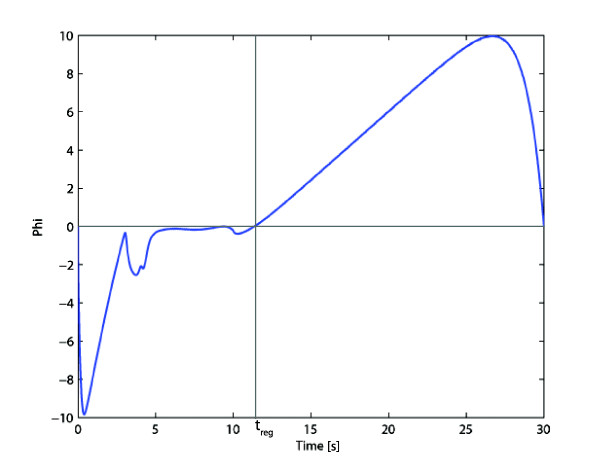
**Plot of *ϕ*(*λ*(*t*), *x*(*t*)) (9)**. This function changes sign at the optimal instant of control switching *T*_*reg*_.

In this case the dependency of the Hamiltonian function on *u *is linear (as given by (8) below). For the prototype considered, $\phi \left(\lambda \left(t\right),\;x\left(t\right)\right)=\left(\lambda_{4}\alpha_{4}x_{3}^{g_{43}}x_{2}^{g_{42}}-\lambda_{3}\alpha_{3}x_{2}^{g_{32}}\right)$. Figure 5 shows a plot of *ϕ*(*λ*(*t*), *x*(*t*)) obtained with a near-optimal control function *u*(*t*). As expected, *ϕ*(*λ*(*t*), *x*(*t*)) is negative for values smaller than *t*_*reg*_, leading to an optimal control *u*(*t*) = 0 and becomes positive for values larger than *t*_*reg*_, leading to *u*(*t*) = 1. Thus, the optimal control is obtained on the extremes of the allowed interval and furthermore, one single switch (from 0 to 1) is enough to achieve the optimal control. It should be remarked that, since *ϕ*(*λ*(*t*), *x*(*t*)) is close to zero around *t *= *t*_*reg*_, in practice, when using a numeric method there can be some jittering in the transition of the manipulated variable.

## Conclusions

For a class of networks in which the yield of the product that favors cell population growth (the "natural" product) competes with the desired product yield, with the manipulated variable affecting linearly the fluxes, it has been shown that the optimal control assumes only extreme values. While the implementation of this optimal control poses no challenge on *in silico *metabolic networks, on real metabolic networks complex bioengineering skills are required. Gene knockout manipulations do not adequate to this kind of control problem due to the long time scale associated with these techniques. The manipulation of specific enzyme levels, controlled by modulating the expression of the corresponding genes using promoter systems and inducers, is a possible solution to this kind of control problem [14].

The use of a bi-level optimization strategy, that maximizes the natural product in the inner level by manipulating the fluxes, leads to a good approximation to the optimal solution, with the advantage of not requiring the full knowledge of the network model. Real networks are extremely complex and exhibit relations between metabolites that are not always expected or fully understood. This gives emphasis to the need of good *in silico *models and also to the determination of the exact branches to be modified when optimizing a network. Although the example network used is very simple, it has proved to be useful to test the optimization strategies but a more complex network should be used to confirm that the strategy can be scaled to a larger network.

## Methods

### The optimization problem

The optimization problem consists in relation to a nonlinear state model of a metabolic network like (2), in selecting *u*(*t*) for *t *∈ [0, *t*_*final*_] such that:

(3) $$J\left(u\right)=x_{\text{5}}\left(t_{final}\right)$$

is maximized under the constraint that *u*(*t*) ∈ [0, 1], ∇*t *≥ 0.

The solution of the optimization problem is obtained using different approaches. Before accomplishing this task, Pontryagin's Maximum Principle is invoked to establish a particular form of the optimal control function for the class of problems at hand.

### Optimization

The control function is now optimized in order to obtain a maximum yield of biomass at the end of the run-time (*t*_*final*_). Three different methods, assuming various levels of information about the network, are considered in order to attain this goal.

The first method, direct optimization, is used as a benchmark to compare the results of the other methods. The last two methods rely on a Bi-level optimization and illustrate a possible solution to the optimization problem when the information about the network is incomplete.

#### Direct optimization

The first method, Direct Optimization, is used mainly as a benchmark, to compare the results of the following methods. Since it is assumed that all the information about the network kinetics is known, the system of differential equations, described in (2) is used. Given a function that receives *t*_*reg *_as input and outputs the final yield of *x*_5_, this optimization tests all the possible values of *t*_reg _and returns the function *J*(*t*_*reg*_) = *x*_5_(*t*_*final*_). The value of *t*_*reg *_that results on a maximum product yield is then determined by solving a simple optimization problem.

The optimization was tested with two MATLAB functions: *fmincon*, from the standard optimization toolbox, that finds the minimum of a constrained nonlinear multi variable function, and *simannealingSB *from Systems Biology Toolbox [17] that performs simulated annealing optimization.

#### Bi-Level Optimization algorithm structure

The Bi-Level optimization algorithm was structured so as to accommodate missing information on the network kinetics. The boxed metabolites and fluxes from Figure 1 are a part of the network that might not be fully described in terms of kinetics. In this approach the missing kinetic information is replaced by stoichiometric data and flux balance analysis is used to obtain the proper flux distribution. Then, an inner optimization determines the fluxes during the batch time. The first step of the inner optimization process is to define the initial conditions of the input *x*_1 _and outputs *x*_3_, *x*_5_. A valid distribution for the fluxes *v*_1_, *v*_2_, *v*_3 _and *v*_4 _is then obtained.

**Figure 6:**
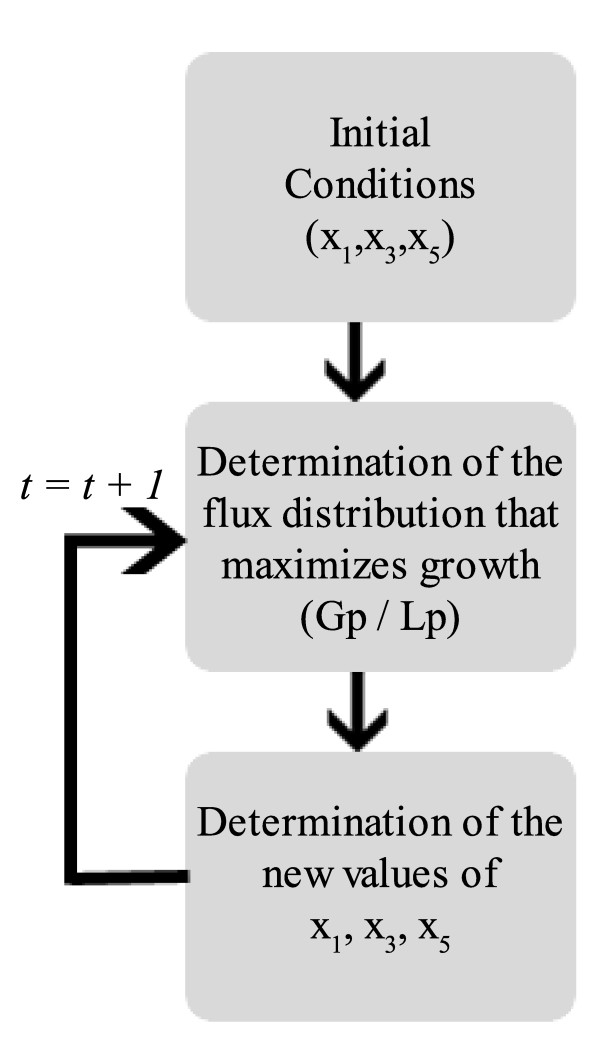
**Inner-Optimization algorithm**. Block diagram of the Inner-Optimization algorithm.

After obtaining the flux distribution, new values for the input/outputs can be calculated by integrating their expressions in the considered time interval. During this time interval the function *u*(*t*) and the values of *v*_1_, *v*_2_, *v*_3 _and *v*_4 _are kept constant. This process is repeated along a time grid from *t *= 0 to *t *= *t*_*final*_. The time interval for the integration was defined to be 1 second. The inner optimization process allows us to obtain the product yield, *x*_5_(*t*_*final*_), given a certain *u*(*t*), taking into account a valid approximation of the network dynamics over the simulation time. The detailed fluxogram of the inner-optimization is shown in Figure 6.

**Figure 7:**
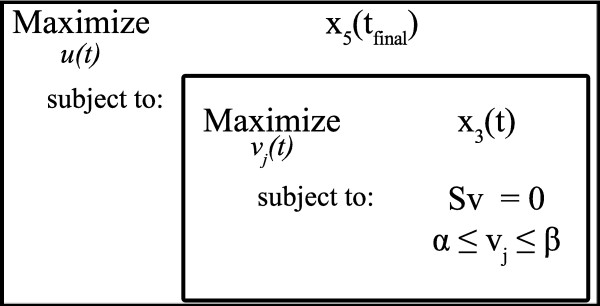
**Bi-Level optimization formulation**. Structure of the Bi-Level optimization.

The bi-level optimization algorithm can be represented schematically as in Figure 7.

#### Inner-optimization using Geometric Programming

On the first implementation of the Bi-Level optimization algorithm the dynamics of the boxed metabolites from Figure 1 are used but, following the algorithm structure, steady-state is assumed. Thus, ${\dot{x}}_{2}$ and ${\dot{x}}_{4}$ from (2) become:

(4) $$\begin{array}{l} \frac{dx_{2}}{dt}=\alpha_{2}x_{1}^{g_{21}}-\beta_{2}x_{3}^{h_{23}}x_{2}^{h_{22}}=0\\ \frac{dx_{4}}{dt}\;=\;\alpha_{4}x_{3}^{g_{43}}x_{2}^{g_{42}}u-\beta_{4}x_{4}^{h_{44}}=0 \end{array}$$

In this algorithm implementation, the inner optimization problem determines the profile of the metabolites, instead of fluxes, due to the nature of the equations. The metabolite concentrations are calculated at the beginning of each time interval, solving a Geometric Programming problem, and used with (2) to integrate the values of *x*_1_, *x*_3 _and *x*_5 _during that interval.

#### Inner-optimization using Linear Programming

On the second implementation it is assumed that only stoichiometric information is available for the reactions inside the box of Figure 1. Assuming steady state, the equations of ${\dot{x}}_{2}$ and ${\dot{x}}_{4}$ become:

(5) $$\begin{array}{l} \frac{dx_{2}}{dt}=v_{1}-v_{2}\times \left(1-u\right)-v_{3}\times u=0\\ \frac{dx_{4}}{dt}\;=\;v_{3}\times u-v_{4}=0 \end{array}$$

Figure 1 shows a regulation from *x*_3 _(Biomass) to flux *v*_3_. Since stoichiometric models do not account for feedbacks, the effect of *x*_3 _can not be integrated directly in the equations. Assuming that the forward feedback leads to an over expression of flux *v*_3_, then a valid solution is to model the forward feedback as a variation of the constraints applied to flux *v*_3_. Setting flux *v*_2 _(precursor of Biomass formation) as the objective function, the FBA problem is solved with the previous equations to obtain a valid and unique flux distribution at each time step. In the context of the inner-optimization, these fluxes are then used to calculate the values of the input/outputs.

#### Pontryagin's Maximum Principle

A general tool to solve dynamic optimization problems such as the one considered here is Pontryagin's Maximum Principle PMP [13].

Let *x *be the state of a dynamical system with control inputs *u *such that:

(6) $$\dot{x}=F\left(x,\;u\right),\;\;\;x\left(0\right)=x_{0},\;\;\;u\left(t\right)\in U,\;\;\;t\in \left[0,\;T\right]$$

where *F *: ℜ^*n *^× ℜ → ℜ^*n*^, *U *is the set of valid control inputs and *T *is the final time, assumed here to be constant.

The control function *u *must be chosen in order to maximize the functional *J*, defined by:

(7) $$J(u)=\psi (x_{i}(T))+\int_{0}^{T}L(x(t),\;u(t))dt$$

Where *ψ *is the cost associated with the terminal condition of the system and *L *the Lagrangian.

According to PMP, a necessary condition for the optimal control is that, along the optimal solution for the state *x*, co-state *λ *and control *u *the Hamiltonian *H *is maximum with respect to *u *[13].

Comparing the cost (3) with the generalized case (7) and taking into consideration that, in the case at hand, given by (1), the dynamics vector field depends linearly on the control, it follows that

(8) H(λ, x, u)=λϕ(λ, x)u

where *ϕ*(*λ*, *x*) is a function that does not explicitly depend on *u*. Since, according to (8), the Hamiltonian is linear in *u*, its maximum is obtained at the boundary of the admissible control set *U*.

Hence, this shows that, for the metabolic network (1), the control that optimizes (3) only assumes the values *u *= 0 or *u *= 1.

In the case at hand, we are interested in maximizing the final value of the state *x*_5_. Since the Lagrangian (L) is zero, (7) becomes *J*(*u*) = *ψ*(*x*_*i*_(*T*)). Thus, the functional *J *to be maximized is:

(9) $$\psi (x(T))=u_{5}(T_{final})$$

as shown before in (3).

Taking into account that, *L *= 0 the adjoint equations are reduced to

(10) $$\dot{\lambda }=-f_{x}^{T}\lambda$$

The network is described by the system of ordinary differential equations in (2), if we consider the state model in the form of *f*(*x*, *u*), where *u *is the control function, calculating *f*_*x*_(*x*, *u*) is straightforward.

Thus

(11) $$\begin{array}{ccc} {\dot{\lambda }}_{1} & = & \beta_{1}h_{11}x_{1}^{h_{11}-1}\lambda_{1}-\alpha_{2}g_{21}x_{1}^{g_{21}-1}\lambda_{2}\\ {\dot{\lambda }}_{2} & = & \begin{array}{l} \beta_{2}x_{3}h^{23}h_{22}x_{2}^{h_{22}-1}\lambda_{2}-\;\;(\alpha_{3}g_{32}x_{2}^{g_{32}-1})\;\;(1-u)\lambda_{3}\\ -\alpha_{4}x_{3}^{g43}ug_{42}x_{2}^{g_{42}}{}^{-1}\lambda_{4} \end{array}\\ {\dot{\lambda }}_{3} & = & \beta_{2}x_{2}^{h_{22}}h_{23}x_{3}^{h_{23}-1}\lambda_{2}-\alpha_{4}ux_{2}^{g}42g_{43}x_{3}^{g_{43}-1}\lambda_{4}\\ {\dot{\lambda }}_{4} & = & \beta_{4}h_{44}x_{4}^{h_{44}-1}\lambda_{4}-\alpha_{5}g_{54}x_{4}^{g_{54}-1}\lambda_{5}\\ {\dot{\lambda }}_{5} & = & 0 \end{array}$$

The terminal conditions for the co-states *λ *are

(12) $$\lambda_{n}(T)=\frac{\partial \psi }{\partial x}{|}_{x=x(T)}\;=([0\;0\;0\;0\;1])$$

Since *L *= 0 the Hamiltonian is given by *λ*^*T *^*f*(*x*).

Substituting in the expression and after some manipulation, becomes:

(13) $$\begin{array}{l} H(\lambda (t),\;x(t),\;u(t),\;t)=\\ \lambda_{1}\left(k-\beta_{1}-x_{1}^{h_{11}}\right)+\lambda_{2}\left(\alpha_{2}x_{1}^{g_{21}}-\beta_{2}x_{3}^{h_{23}}x_{2}^{h_{22}}\right)+\lambda_{5}(\alpha_{5}x_{4}^{g_{54}})+\\ \lambda_{3}\alpha_{3}x_{2}^{g_{32}}-\alpha_{4}\beta_{4}x_{4}^{h_{44}}+(\lambda_{4}\alpha_{4}x_{2}^{g_{43}}x_{2}^{g_{42}}-\lambda_{3}\alpha_{3}x_{2}^{g_{32}})u \end{array}$$

that depends linearly on the control function *u*, as expected.

The derivative of the Hamiltonian in order to the control function is:

(14) $$\begin{array}{ccc} H_{u} & = & \lambda^{T}f_{u}\\ & = & -\lambda_{3}\alpha_{3}x_{2}^{g_{32}}+\lambda_{4}\alpha_{4}x_{3}^{g_{43}}x_{2}^{g_{42}} \end{array}$$

This expression is the one that determines the number of switches between 0 and 1 of the control variable.

## Authors' contributions

AD helped in the research of the state of the art, implemented the software and drafted the manuscript. SV was involved in the creation and modeling of the prototype network, formulation of the optimization processes and helped to draft the manuscript. JML provided the mathematical basis for the optimization and control techniques and helped to draft the manuscript. All authors read and approved the final manuscript.

## References

[B1] Nielsen J Metabolic engineering Appl Microbiol Biotechnol 2001 55 3 263 83 10.1007/s002530000511 11341306

[B2] Chu WB Constantinides A Modeling, optimization, and computer control of the cephalosporin C fermentation process Biotechnol Bioeng 1988 32 3 277 88 [Chu, W B Constantinides, A United States Biotechnology and bioengineering Biotechnol Bioeng. 1988 Jul 20;32(3):277-88.] 10.1002/bit.260320304 18584748

[B3] Rocha I Maia P Evangelista P Vilaca P Soares S Pinto JP Nielsen J Patil KR Ferreira EC Rocha M OptFlux: an open-source software platform for in silico metabolic engineering BMC Syst Biol 4 45 10.1186/1752-0509-4-45 20403172 PMC2864236

[B4] Edwards JS Covert M Palsson B Metabolic modelling of microbes: the flux-balance approach Environ Microbiol 2002 4 3 133 40 10.1046/j.1462-2920.2002.00282.x 12000313

[B5] Varma A Palsson BO Stoichiometric flux balance models quantitatively predict growth and metabolic by-product secretion in wild-type Escherichia coli W3110 Appl Environ Microbiol 1994 60 10 3724 31 7986045 10.1128/aem.60.10.3724-3731.1994 PMC201879

[B6] Schilling CH Edwards JS Letscher D Palsson BO Combining pathway analysis with flux balance analysis for the comprehensive study of metabolic systems Biotechnol Bioeng 2000 71 4 286 306 10.1002/1097-0290(2000)71:4<286::AID-BIT1018>3.0.CO;2-R 11291038

[B7] Llaneras F Pico J Stoichiometric modelling of cell metabolism J Biosci Bioeng 2008 105 1 11 [Llaneras, Francisco Pico, Jesus Research Support, NonU.S. Gov't Review Japan Journal of bioscience and bioengineering J Biosci Bioeng. 2008 Jan;105(1):1-11.] 10.1263/jbb.105.1 18295713

[B8] Burgard AP Pharkya P Maranas CD Optknock: a bilevel programming framework for identifying gene knockout strategies for microbial strain optimization Biotechnol Bioeng 2003 84 6 647 57 10.1002/bit.10803 14595777

[B9] Rocha M Maia P Mendes R Pinto JP Ferreira EC Nielsen J Patil KR Rocha I Natural computation meta-heuristics for the in silico optimization of microbial strains BMC Bioinformatics 2008 9 499 10.1186/1471-2105-9-499 19038030 PMC2612012

[B10] Koh K Kim S Mutapic A Boyd S GGPLAB: A simple Matlab toolbox for Geometric Programming 2006

[B11] Boyd SP Vandenberghe L Convex Optimization 2004 Cambridge University Press

[B12] Marin-Sanguino A Voit EO Gonzalez-Alcon C Torres NV Optimization of biotechnological systems through geometric programming Theor Biol Med Model 2007 4 38 10.1186/1742-4682-4-38 17897440 PMC2231360

[B13] Lewis F Syrmos V Optimal Control 1995 2 John Wiley & Sons Inc. New York

[B14] Kapil G Gadkar RM III F JD Optimal genetic manipulations in batch bioreactor control Automatica 2006 42 10 1723 1733 10.1016/j.automatica.2006.05.004

[B15] Gaspar P Neves AR Ramos A Gasson MJ Shearman CA Santos H Engineering Lactococcus lactis for Production of Mannitol: High Yields from Food-Grade Strains Deficient in Lactate Dehydrogenase and the Mannitol Transport System Appl Environ Microbiol 70 15006767 10.1128/AEM.70.3.1466-1474.2004 PMC368346

[B16] Sorribas A Hernandez-Bermejo B Vilaprinyo E Alves R Cooperativity and saturation in biochemical networks: a saturable formalism using Taylor series approximations Biotechnol Bioeng 2007 97 5 1259 77 10.1002/bit.21316 17187441

[B17] Schmidt H Jirstrand M Systems Biology Toolbox for MATLAB: a computational platform for research in systems biology Bioinformatics 2006 22 4 514 515 10.1093/bioinformatics/bti799 16317076

